# Implantable collamer lens surgery in patients with primary iris and/or ciliary body cysts

**DOI:** 10.1186/s12886-018-0935-7

**Published:** 2018-11-06

**Authors:** Zhen Li, Zhike Xu, Yaqin Wang, Qiang Liu, Bin Chen

**Affiliations:** Department of Ophthalmology, The People’s Hospital of Leshan, 635 Wanghaoer Street, Leshan, Sichuan Province 614000 People’s Republic of China

**Keywords:** Implantable collamer lens, Myopia, Phakic intraocular lens, Primary iris and ciliary body cyst, Ultrasound biomicroscope

## Abstract

**Background:**

The prevalence of primary iris and/or ciliary body cysts is common in myopia, though asymptomatic in nearly all cases. It’s a very valuable thing to study the clinical safety and reliability of implantable collamer lens (ICL) surgery in patients with primary iris and/or ciliary body cysts.

**Methods:**

A total of 108 patients (201 eyes) were included in this retrospective study. All eyes had been implanted with V4c implantable collamer lens (ICLV4c). According to the eyes with or without primary iris and/or ciliary body cysts, all eyes were divided into two groups. We observed preoperative and postoperative uncorrected distance visual acuity (UDVA), corrected distance visual acuity)(CDVA), intra-ocular pressure(IOP), anterior chamber volume(ACV), anterior chamber depth(ACD), trabecular-iris angle (TIA), angle opening distance at 500 μm (AOD500),vertical central distance between the corneal endothelium and the front surface of ICL(CE-ICL), and the central vault. The follow-up periods covered 12 months.

**Results:**

Among all the 201 eyes, primary iris and/or ciliary body cysts were detected in 54 eyes (26.87%),but the prevalence was account to 36.11%(18males,21females).There were 30 eyes (55.56%) with unilateral single cyst, 12 eyes (22.22%) with unilateral double cysts, 12 eyes (22.22%) eyes with unilateral multiple and/or multi-quadrants cysts, the mean size of cysts was (0.714 ± 0.149)mm(range from 0.510 to 1.075 mm).30.4% of the cysts were located at iridociliary sulcus, 65.5% in pars plicata, and 4.1% in midzonal iris, which showed a characteristic distribution pattern, with cysts found predominantly in the inferior and temporal quadrants.The postoperative size and the number of cysts showed nearly no changes. The postoperative ACV, AOD500 and TIA showed a statistical reduction in both two groups (*P* < 0.05), but with no statistical significant between the two groups (*P* > 0.05), the parameters of postoperative IOP,CE-ICL and central vault also showed the same results as which. We did not observe serious complication and IOP elevating in the whole follow-up periods.

**Conclusion:**

Primary iris and/or ciliary body cysts are not absolutely contraindication for ICL surgery. For some single cyst smaller than 1.075 mm or single quadrant cysts located at ciliary body are rare to lead some serious complications. But, for some multiple cysts, especially multi-quadrants cysts located at iridociliary sulcus, we still should remain cautions.

## Background

The implantable collamer Lens (ICL) (STAAR Surgical Co.), which have been reported to perform well for the correction of moderate to high myopia [1–4]. But the prevalence of primary iris and/or ciliary body cysts in myopia is common, especially in young adult or middle-aged women [5]. In the last years, primary iris and /or ciliary body cysts were hardly detected by using common ophthalmic examinations. With the development of advanced medical technique, UBM is an important investigation which could effectively excludes the differential diagnosis of some ring melanoma of the iris and multiple separate cysts of the iris pigment epithelium [6]. Primary iris and/or ciliary body cysts are usually located inferotemporally in the anterior segment, most commonly in the iridociliary sulcus [7].Though asymptomatic in nearly all cases, they may rarely enlarge or cause secondary glaucoma, corneal decompensation [8]. Because, there were no relative reports about the safety and reliability of ICL implantation in patients with iris and/or ciliary body cysts. Our research was aimed to evaluate the clinical safety and reliability of ICL surgery in patients with primary iris and/or ciliary body cysts.

## Methods

### Patient and public involvement

Our study was a retrospective analysis. A total of 108 patients (201 eyes) were included in this study. All eyes were implanted with the myopic ICLV4c (ICMV4C model) by an experienced doctor at the Department of ophthalmology, The People’s Hospital of Leshan, Sichuan Province, China, from July 2015 to December 2016. Group1 included 54 eyes with primary iris and/or ciliary body cysts. Group2 included 147 eyes without cysts. Before our analysis, all patients and /or public were not involved in the design of this study. This project was approved by the science and technology foundation of Sichuan Provincial Health and Family Planning Commission (NO.150065), which met the demand of Declaration of Helsinki. Informed consents were obtained from all subjects.

### Data collection

Inclusion criteria for this study included patients age between 18 and 45 years, myopia between --2.50 and − 21.00DS, astigmatism between 0 and − 6.00 DC, anterior chamber depth (ACD) of 2.80 mm or more, and an endothelial cell density greater than 2000 cells/mm^2^. Patients were also required to have a reasonable expectation of surgical outcomes, no preexisting ocular pathology, no keratoconus, cataract or glaucoma, and no serious systemic diseases [9]. Preoperatively, all the patients underwent a complete ophthalmic examination,which included uncorrected distance visual acuity (UDVA), corrected distance visual acuity(CDVA), slit lamp biomicroscopy, indirect ophthalmoscopy, corneal thickness (CT), anterior chamber volume (ACV) (Pentacam, Oculus,Germany), intra-ocular pressure (IOP), the horizontal white-to-white distance, endothelial cell density, anterior chamber depth (ACD),trabecular-iris angle (TIA) and angle opening distance at 500 μm (AOD500). ACD was measured from the endothelial surface of cornea to the anterior surface of lens. TIA was measured with the apex in the iris recess and the arms of the angle passing through a point on the trabecular meshwork from the scleral spur and the point on the iris perpendicularly opposite.AOD500 was the distance between the posterior cornea surface and the anterior iris surface measured on a line perpendicular to the trabecular meshwork at 500 mm from the scleral spur [10]. Gonioscopy showed an open anlge all over in both eyes. The follow-up periods covered 12 months. By the end of the 1st,the 3rd,the 6th and the 12th month postoperatively, we observed the vertical central distance between the corneal endothelium and the front surface of ICL (CE-ICL), central vault, IOP, AOD500, TIA,ACV, endothelial cell density,UDVA and CDVA. AOD500 and TIA were measured by UBM, ACV by Pentcam. Endothelial cell density was determined using a noncontact specular microscope by one single operator (J.Y). (SP-8800, Konan, Nishinomiya, Japan).All image acquisitions were operated by the same physician. The central vault was defined as the distance between the back surface of the ICL and the anterior crystalline lens pole. The UDVA and CDVA were checked using Snellen charts and converted to the logMAR scale for statistical analysis.

### Ultrasound biomicroscopy

**Fig. 1:**
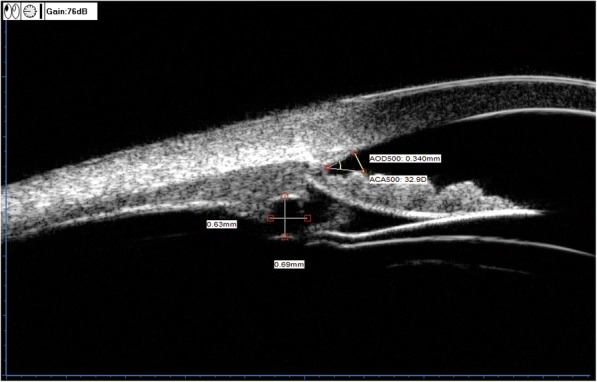
Postoperative parameters of TIA and AOD500 corresponding to the cysts on the UBM image

ACD, TIA,CE-ICL,AOD500,central vault were performed using a high-resolution UBM (SW-3200, SUOER, China) with a 50–100 MHz transducer-probe. All procedures were performed by the same experienced examiner in constant ambient lighting conditions. Any cysts detected in UBM were recorded for the size (the horizontal and vertical diameters in radial position), location, clock position, corresponding AOD500 and TIA. The parameters of AOD500 and TIA in 3, 6, 9, 12’o clock were separately recorded. By the end of the 6th month postoperatively, the mean value of TIA and AOD500 in 3, 6, 9, 12’o clock was separately considered to statistical analysis. Anterior chamber angle was considered to be closed on UBM image if any contact between the iris and angle wall anterior to the scleral spur. The anterior chamber angle corresponding to the cysts, which is defined as the anterior chamber angle with cyst in the largest vertical diameter on UBM image, was further assessed by AOD500 and TIA (Fig. 1). According to the distribution of cysts, single quadrant cysts were defined as a distribution of the cysts equal to or less than one quadrant, multi-quadrant cysts were defined as cysts in more than one quadrant. According to the number of cysts, single cyst was defined as only one cyst found within the iris and/or ciliary body, double cysts were defined as 2 cysts, and multiple cysts were defined as equal to or more than 3 cysts [11].

### ICL surgical procedure

After topical anaesthesia (0.4% oxybuprocaine hydrochloride, Santen, Japan) and injection f a viscoelastic surgical agent (1.7% Sodium hyaluronate; Bausch & Lomb, China) into the anterior chamber, an ICLV4c was inserted via a 3.0 mm temporal clear corneal incision with the use of an injector cartridge (STAAR Surgical). After the ICL was placed in the posterior chamber, the surgeon then completely removed the viscoelastic surgical agent from the eye using a balanced salt solution. All surgeries were uneventful and no intra-operative complications were observed. Following surgery, a combination antibacterial and steroidal medication (0.1% Tobramycin dexamethasone, Alcon, China) was prescribed four times daily for 2 weeks.

### Statistical analysis

Statistical analysis was performed by using SPSS 20.0. Parametric and nonparametric tests were used to compare continuous variables, according to data distribution. For nonparametric data, Kolmogorov-Smirnov test was applied for all variables and resulted in nonsignificant outcomes indicating the normality of data distribution. The Mann–Whitney U test was used to analyze the differences between the two groups. Differences in mean values of preoperative and postoperative ocluar biometric parameters within one group were examined using a paired Student’s *t*-test. *P* value less than 0.05 was considered significant statistically.

## Results

A total of 108 patients (201 eyes) were included in this retrospective study. Preoperative parameters showed no statistical difference between the two groups in the terms of biometric data, UDVA, CDVA, IOP, ACD, ACV, AOD500, TIA and CT (Table 1). There were no intra-operative and postoperative ocular or systemic complications.

**Table 1:** Preoperative ocular parameters in two groups (Mean ± _ Standard Deviation)

Parameters	Groups			
	Group1(Mean ± SD)	Group 2(Mean ± SD)	t	*P*
UDVA	0.04 ± 0.03	0.03 ± 0.03	1.460	0.149
CDVA	0.91 ± 0.18	0.78 ± 0.25	1.948	0.056
Manifest refractive sphere(D)	12.76 ± 4.00	13.71 ± 5.01	−0.723	0.427
Manifest refractive cylinder(D)	1.78 ± 1.13	1.44 ± 0.92	1.235	0.221
IOP	15.33 ± 3.76	16.14 ± 3.13	−0.889	0.377
ACD	3.25 ± 0.19	3.16 ± 0.24	1.422	0.160
CT	528.61 ± 37.98	526.06 ± 28.31	0.297	0.797
ACV	216.00 ± 30.07	199.90 ± 30.57	1.938	0.057
AOD500	0.617 ± 0.222	0.583 ± 0.185	0.639	0.525
TIA	47.92 ± 9.79	47.40 ± 9.43	0.199	0.843

### Primary iris and ciliary body cysts

**Fig. 2:**
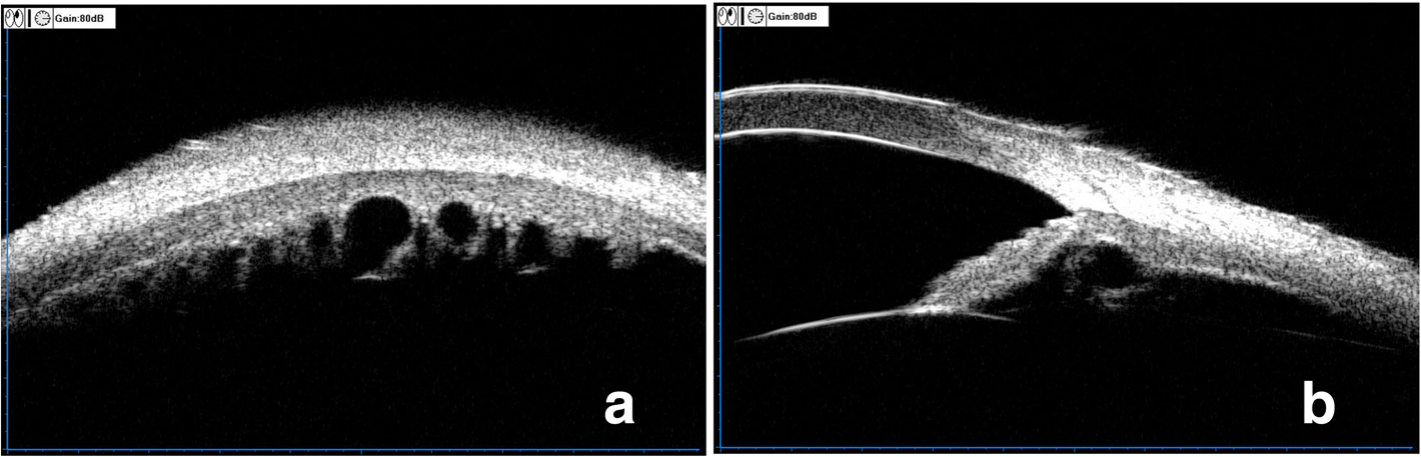
Preoperative vertical and horizontal scanning images of multiple cysts. **a**. Preoperative vertical scanning image of multiple cysts in 3’o clock in left eye. **b**. Preoperative horizontal scanning image of multiple cysts in 3’o clock in left eye

Among all the 201 eyes, primary iris and/or ciliary body cysts were detected in 54 eyes (26.87%), but the prevalence of cysts in patients was account to 36.11% (18males,21females). Among all the eyes with cysts, there were 30 eyes (55.56%) with unilateral single cyst, 12 eyes (22.22%) with unilateral double cysts, 12 eyes (22.22%) eyes with unilateral multiple and/or multi-quadrants cysts, the mean size of cysts was (0.714 ± 0.149)mm(range from 0.510 to 1.075 mm).30.4% of the cysts were located at iridociliary sulcus, 65.5% at pars plicata, and 4.1% at midzonal ris, which showed a characteristic distribution pattern, where cysts found predominantly in the inferior and temporal quadrants. Postoperatively, the size and the number of cysts showed nearly no changes. Figure 2a and b showed a 31-year-old patient in her left eye with multiple and multi-quadrant cysts.

### Change of anterior chamber parameters

We observed the change of anterior chamber according to the parameters, which included the ACV, AOD500 and TIA. Table 2 showed the preoperative mean AOD500, TIA corresponding to the cysts and the mean value of AOD500, TIA in four clocks. Statistical analysis showed no statistical significant (*P* > 0.05). The range of AOD500 was 0.183 mm to 1.129 mm corresponding to the location of cysts, while the degree of TIA was 20.20° to 64.70°. Table 3 showed the preoperative and postoperative parameters of AOD500 and TIA in 3, 6, 9, 12’o clock. By the end of 6th month postoperatively, all the parameters of AOD500 and TIA in 3, 6, 9, 12’o clock were proved to be significant reduced in both two groups (*P* < 0.05). But, the preoperative and postoperative parameters of AOD500 and TIA in 3, 6, 9, 12’o clock showed no statistical significant between the two groups (*P* > 0.05).The postoperative parameters of ACV also showed the same reduction as AOD and TIA in both two groups (*P* < 0.05), but with no statistical significant between the two groups (*P* > .005).

**Table 2:** Comparison of the preoperative TIA and AOD500 corresponding to the cysts (Mean ± _ Standard Deviation)

	Location			
	Corresponding to the cysts (Mean ± SD)	mean value of 4 clocks (Mean ± SD)	t	*P*
AOD500	0.573 ± 0.202	0.617 ± 0.222	0.719	0.476
TIA	47.17 ± 10.75	47.92 ± 9.79	0.818	0.807

**Table 3:** Comparing the parameters of AOD500 and TIA in 3,6,9,12’o clock

Location Group and *P*-value		TIA(°)			AOD500(mm)		
		Pre-operation post-operation(6th month)		*P*	Pre-operation post-operation (6th month)		*P*
3’o clock	Group1	45.40 ± 8.29	22.67 ± 6.15	0.000	0.51 ± 0.14	0.21 ± 0.06	0.000
	Group2	47.37 ± 8.29	27.56 ± 8.50	0.000	0.53 ± 0.13	0.26 ± 0.09	0.000
	P-value	0.560	0.368		0.750	0.314	
6’o clock	Gropu1	40.77 ± 12.98	24.11 ± 4.27	0.000	0.46 ± 0.20	0.21 ± 0.04	0.001
	Group2	52.71 ± 7.87	21.60 ± 2.97	0.013	0.68 ± 0.23	0.19 ± 0.03	0.009
	P-value	0.090	0.368		0.101	0.633	
9’o clock	Group1	44.96 ± 9.00	26.30 ± 7.11	0.001	0.53 ± 0.19	0.25 ± 0.09	0.001
	Group2	48.03 ± 4.53	25.54 ± 5.72	0.000	0.56 ± 0.09	0.26 ± 0.06	0.001
	P-value	0.314	1.000		0.315	0.832	
12’o clock	Group1	42.96 ± 9.15	20.84 ± 5.80	0.000	0.48 ± 0.19	0.19 ± 0.05	0.000
	Group2	45.16 ± 4.41	19.67 ± 4.53	0.000	0.52 ± 0.08	0.19 ± 0.06	0.002
	P-value	0.289	0.711		0.20	0.791	

### Visual outcomes

The postoperative UDVA was proved to be significantly improved for both two groups (*p* < 0.05), but, which showed no statistical significant between the two groups in follow-up periods (*P* > 0.05).

### IOP, vault and CE-ICL

Table 4 showed the change of IOP, central vault and CE-ICL, which showed no statistical significant between the two groups in follow-up periods (*P* > 0.05).

**Table 4:** Postoperative parameters of IOP, Vault and CE-ICL for the two groups

Parameters	Time	Groups		
		Group 1(Mean ± SD)	Group2(Mean ± SD)	1P
IOP(mmHg)	1mo	17.11 ± 2.95	16.20 ± 3.25	0.295
	3mo	16.83 ± 2.04	17.27 ± 2.62	0.578
	6mo	16.33 ± 2.74	16.71 ± 2.64	0.560
	12mo	16.44 ± 1.98	16.78 ± 2.35	0.580
2 P	P > 0.05			
Vault(mm)	1mo	0.62 ± 0.26	0.50 ± 0.27	0.157
	3mo	0.62 ± 0.25	0.52 ± 0.24	0.197
	6mo	0.62 ± 0.25	0.51 ± 0.23	0.111
	12mo	0.61 ± 0.23	0.51 ± 0.22	0.109
2P	P > 0.05			
CE-ICL (mm)	1mo	2.23 ± 0.32	2.37 ± 0.37	0.132
	3mo	2.25 ± 0.27	2.35 ± 0.33	0.243
	6mo	2.25 ± 0.26	2.34 ± 0.33	0.264
	12mo	2.26 ± 0.24	2.35 ± 0.33	0.229
2P	P > 0.05			

## Discussion

**Fig. 3:**
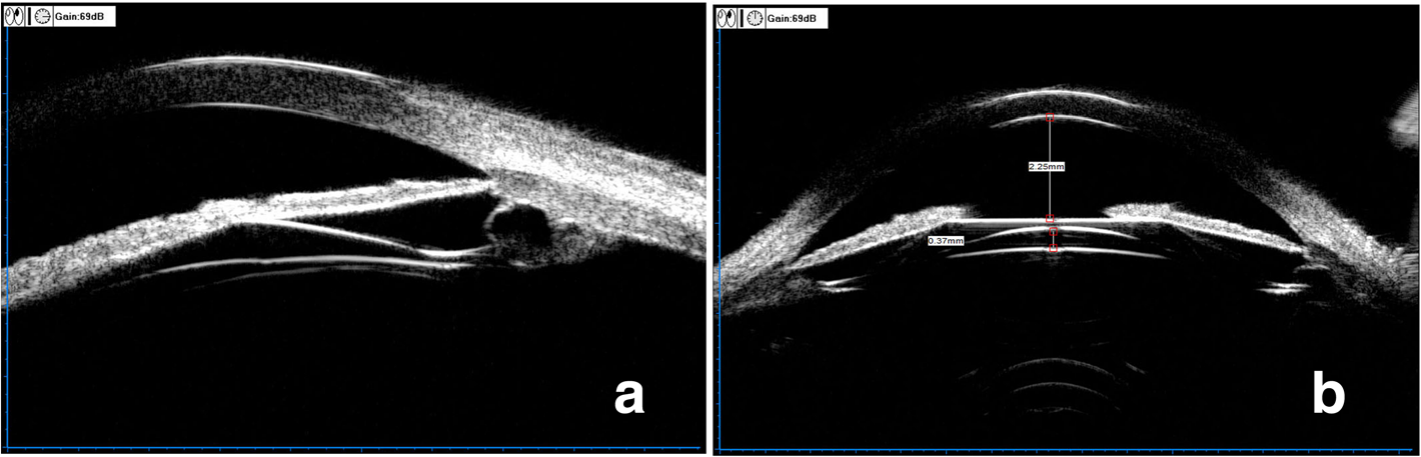
Postoperative horizontal scanning image of multiple cysts in 3’o clock in left eye. **a**. The haptics of ICL would induce the broken of cyst capsule. **b**. The haptics of ICL in the location of cysts in ciliary sulcus, which would be likely to push the root of iris forwarding and induce the anterior chamber angle narrow or close up

**Fig. 4:**
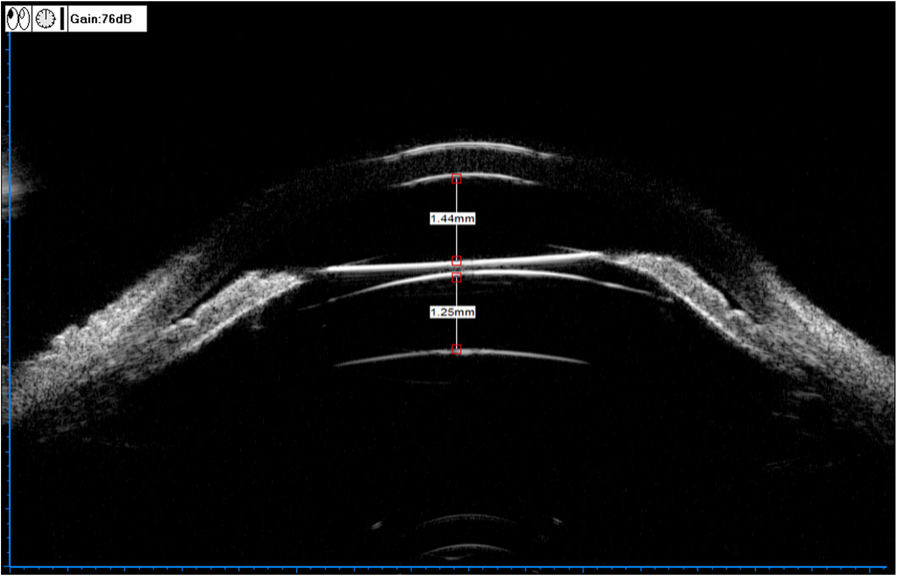
Excessive vault is a high risk factor to induce glaucoma. In this eye, the central vault was more than 1.25 mm, and the anterior chamber angle was closed in the location of ICL haptics

Compared with custom corneal laser refractive surgery or clear lens extraction surgery, the ICL surgery has been proved to have many potential advantages, including the possibility of correction of high refractive errors, maintaining the integrity of the corneal structure and accommodation of the lens, and the reversibility [3, 4]. In our study, all cases in both two groups finally gained good UDVA and also showed no serious complications, which means primary iris and/or ciliary body cysts had no direct impact on the visual outcome in ICL surgery. The diagnosis of primary iris and/or ciliary body cysts was made according to the patients’ history and clinical manifestation, after excluding other possible etiological factors that can cause secondary cysts. Most of primary iris and/or ciliary body cysts are located at the iridociliary sulcus and/or pars plicata, which mainly arise from the iris pigment epithelium [8]. In our study, we still found that almost all cysts were located at the iridociliary sulcus or pars plicata, and were predominantly distributed in the inferior and temporal quadrants. These results were well according with the finding of Kunimatsu [6]. The prevalence of primary cysts was amount to 36.11%, which was higher than 34.4% found by Wang et al. [12]. It was reported that iris-ciliary cysts were the main cause of glaucoma and closure glaucoma [13–15] .If the patients with cysts had been implanted with ICL, the potential risk would include: (1) cysts stimulated ciliary body, causing an increase in aqueous fluid;(2) mucus and other materials produced by cysts deposited trabecular meshwork, thus preventing aqueous fluid from flowing out; (3) If the haptics of ICL just were located at the position of cysts, which might be more likely to push the root of iris forwarding and lead the anterior chamber angle becoming narrowed or closed up**;**(4) The haptics of ICL might cause the broken of cyst capsule, the fluid of cysts might cause serious inflammation (Fig. 3a, b). But, in the whole follow-up periods, we did not find an elevating of IOP in the eyes with cysts. The size of cysts also showed no changes. According to the study of Wang et al. [11], the cysts larger than 0.8 mm or located at iridociliary sulcus were inclined to narrow or close the corresponding angles. When compared with the eyes without cysts, the eyes with multiple and/or multi-quadrants cysts would be more likely to narrow or close the whole anterior chamber angle. But, the eyes only with unilateral single or double cysts did not show the same result. In our study, all the postoperative parameters of AOD500, ACV and TIA in 3, 6, 9, 12’o clock were proved to be significant reduced in both two groups. But, the difference between the two groups showed no statistical significant. The result also showed that the unilateral single cyst or single quadrant cysts would have no significant effect on narrowing or closing anterior chamber angle in ICL surgery. As Zhu’s [16] study, he found significant correlation between high intraocular pressure and the presence of iris-ciliary cysts, particularly the quantity and the size of the cysts. We sincerely suggest that ophthalmologists should monitor the changes of IOP and anterior chamber angle for extended follow-up periods, once the larger primary iris-ciliary cysts or multiple cysts, especially multi-quadrants cysts located at iridociliary sulcus were detected. Central vault is of crucial value for estimating the safety of ICL surgery. Excessive vault is one of risk factors to induce secondary glaucoma, and insufficient vault is responsible for the formation of anterior subcapsular cataract [17, 18]. Previous studies had defined an excellent vault was 250 to 750 *μ*m [19]. In our study, we found that the postoperative mean vault and CE-ICL had no statistical significant between two groups. As we all known that the changes of central vault are mainly according to the difference between the size of ICL and sulcus-to-sulcus (STS) diameter, which would explain why primary iris and/or ciliary body cysts had no direct impact on the change of central vault. In our study, we found the postoperative anterior chamber angle was closed in 3’o clock positions in one eye without cysts. Form the images of UBM (Fig. 4), we observed that the central vault was more than 1.25 mm, the anterior chamber angle was closed at the location corresponding to the ICL haptic. Excessive vaults would induce secondary glaucoma, owe to persistent angle closure, pupil blocking, or pigmentary dispersion [20–24].For all eyes, we did not find an elevating IOP in follow-up periods. We considered, whether the secondary angle closure glaucoma would be happen, which was decided by the range of closed anterior chamber angle. If only one clock position or less than one quarter anterior chamber angle was closed, the secondary glaucoma would not be happen. Besides, with a hole in the central of the lens, which reduce the risk of papillary block.

## Conclusion

In conclusion, primary iris and/or ciliary body are not absolutely contraindication for ICL surgery. For some single cyst smaller than 1.075 mm or single quadrant cysts located at ciliary body are rare to lead some serious complications. But, for some multiple cysts, especially multi-quadrants cysts located at iridociliary sulcus, we still should remain cautions. In the future study, we would collect more samples and monitor longer follow-up periods to prove the safety and reliability of ICL surgery in patients with primary iris and/or ciliary body cysts.

## Abbreviations

ACD: Anterior chamber depth
ACV: Anterior chamber volume
AOD500: Angle opening distance at 500 μm
BCVA: Best corrected visual acuity
CDVA: Corrected distance visual acuity
CE-ICL: Vertical central distance between the corneal endothelium and the front surface of ICL
CT: Corneal thickness
D: Dioptometer
ICL: Implantable collamer lens
IOP: Intraocular pressure
TIA: Trabecular-iris angle
